# Boosting the social impact of innovative cancer research – towards a mission‐oriented approach to cancer

**DOI:** 10.1002/1878-0261.12464

**Published:** 2019-02-27

**Authors:** Ulrik Ringborg, Julio E. Celis, Michael Baumann, Alexander Eggermont, Christopher P. Wild, Anton Berns

**Affiliations:** ^1^ Cancer Center Karolinska Karolinska University Hospital Stockholm Sweden; ^2^ European Academy of Cancer Sciences Danish Cancer Society Research Centre Copenhagen Denmark; ^3^ German Cancer Research Center (DKFZ) Heidelberg Germany; ^4^ German Cancer Consortium (DKTK) Heidelberg Germany; ^5^ Gustave Roussy Cancer Campus Grand Paris Villejuif France; ^6^ International Agency for Research on Cancer (IARC/WHO) Lyon France; ^7^ Oncode Institute The Netherlands Cancer Institute Amsterdam The Netherlands

**Abbreviations**
CCC: comprehensive cancer centre
EC: European Commission
ECPC: European Cancer Patient Coalition
HRQL: health‐related quality of life

The increasing cancer burden is one of the leading medical societal challenges today. The number of new cancer patients in Europe is expected to increase from 3.6 (2015) to 4.3 million over the next two decades; it has now reached 3.91 million. Moreover, the number of patients living with a cancer diagnosis is ever increasing, making cancer a significant chronic disease. In Europe, the total number of cancer deaths amounts to 1.94 million annually (Ferlay *et al*., 2018). So far, the results of our research efforts and their implementation in the healthcare system have been unable to curb this development.

Analyses of European cancer research during the last 16 years by the cancer community and the European Commission (EC) have identified significant barriers hindering the translation of discoveries into preventive measures and therapeutic applications with a direct impact for patients. The complexity of cancer, exacerbated by the heterogeneity of tumours and the existence of a large number of subgroups, calls for new research strategies. Securing a critical mass of expertise with access to complex infrastructures, adequate access to patients, and resources for sustainable activities, as well as more effective coordination of international research efforts, are some of the issues that need urgent attention. Significant recent developments, however, such as the establishment of Cancer Core Europe for therapeutic research (Calvo *et al*., 2018; Eggermont *et al*., 2014) and Cancer Prevention Europe (Forman *et al*., 2018) – both of which are consortia built on institutional collaborations – have set the agenda for tackling these problems in a concerted way. More or less synchronously, the EC recently proposed a mission‐oriented approach to deal with significant societal challenges and pointed out that missions should have a scientific, social, and economic impact (http://europa.eu/rapid/press-release_IP-18-4041_en.htm).

At the request of the EC, JE Celis and D Pavalkis outlined a mission‐oriented approach to cancer (Celis and Pavalkis, 2017) rooted in the efforts of the cancer community, the Commission, patient organizations, and policymakers during the last two decades to attempt to structure cancer research in a manner that would provide patients with the therapeutics and diagnostics that they rightly deserve. Now that a mission‐oriented policy is currently under discussion for Horizon Europe, the cancer community has begun to coordinate their efforts to help realize a mission that could change the lives of many within Europe and beyond.

Towards this end, this Special Issue highlights the essential components needed for a mission‐oriented approach to cancer, and how they should be connected to develop a coherent cancer research continuum stimulating science‐driven and social innovations that impact society at large across Europe and beyond.

The first contribution by Celis and Heitor provides a historical account of the steps leading to a potential cancer mission in Horizon Europe and highlights the value of building communities to address significant challenges in partnership. They argue that new paradigms and conditions for responsible science and innovation policy across the EU require (i) the collective action of Research & Development institutions; (ii) a system approach to health systems, higher education, and patient organizations; and (iii) new initiatives to encourage international cooperation across an enlarged Europe. Sharing expertise, resources, patients, and data; providing evidence‐based advice to inform policy; and interacting closely with policymakers are deemed essential for promoting innovations in the areas of both prevention and health care, which in turn are necessary for achieving the goal of ensuring a long life expectancy for three out of four cancer patients by 2030 (Celis and Heitor, 2019).

The next contribution is a report of the Vatican Conference on ‘A mission‐oriented approach to cancer in Europe: boosting the social impact of innovative cancer research’ that was jointly organized by the European Academy of Cancer Sciences and the Pontifical Academy of Science in November 2018. Both the presentations and the discussions indicated that the cancer community is united, well‐motivated, and prepared to develop a mission‐based approach to cancer benefitting all member states (European Academy of Cancer Sciences, 2019).

Ringborg begins a series of discussions on research issues by defining translational cancer research as a coherent cancer research continuum and proceeds to highlight the need to bridge research components in therapeutics and prevention to facilitate/accelerate the conversion of research discoveries into clinical/prevention applications. Spanning the cancer research/care continuum is deemed essential to a successful mission (Ringborg, 2019).

Eggermont *et al*. present Cancer Core Europe, a legal, bottom‐up consortium of seven large cancer research centres – most of them Comprehensive Cancer Centres (CCCs) – from different countries, which are committed to sharing expertise, resources, patients, and information. Cancer Core Europe has become a model to link basic/preclinical research with early clinical research, while at the same time creating the critical mass needed for developing personalized/precision cancer medicines (Eggermont *et al*., 2019).

Both therapeutics and prevention are needed to tackle the increasing cancer problem, and therefore, Cancer Prevention Europe, a consortium of 10 cancer research centres, was recently established to structure and strengthen prevention research. Wild *et al*. (2019) describe the consortium and highlight its potential to contribute to effective cancer control.

Institutional collaborations at the national level are of fundamental importance for a cancer mission, and Joos *et al*. report on the German Cancer Consortium, a national consortium linking eight CCCs under the leadership of the German Cancer Research Center. This Consortium participates both in Cancer Core Europe and Cancer Prevention Europe, and provides a model for building national/international research collaborations/networks for translational cancer research (Joos *et al*., 2019).

Pan European collaborations are mandatory for achieving critical mass and infrastructure support, and Berns reports on current methodologies for the designation of CCCs and also on a programme for Designation of CCCs of excellence. Consortia consisting of quality assured research centres are needed to cover the most critical research areas within the cancer research continuum (Berns, 2019).

New clinical trial methodologies are necessary considering the large number of tumour subgroups. Moreover, there is a steady improvement in stratification technologies and follow‐up of molecular tumour markers during treatment. Garralda *et al*. (2019) summarize the development of next‐generation clinical trials for the advancement of personalized/precision cancer medicine.

Lacombe *et al*. discuss information from clinical research on new anticancer agents that is needed for adoption by healthcare systems. Currently, conducting randomized comparative clinical trials for assessing clinical efficacy is increasingly complex, and new strategies for drug development are proposed with the aim of providing healthcare systems with relevant information (Lacombe *et al*., 2019).

Primary prevention aims at reducing the causes of cancer and is often based on information on risk or protective factors. According to Schütz *et al*., around half of cancers could be prevented if such information was translated into effective interventions. However, currently, there is a need for greater emphasis both on developing preventive interventions and understanding how best to implement those available into health systems. For some cancers, primary prevention is not possible due to a lack of understanding of the disease aetiology, and here, further research into the identification of risk factors is required (Schütz *et al*., 2019).

A specific type of primary prevention is therapeutic (medical) prevention, previously termed chemoprevention. High‐risk individuals receive medical treatment with the aim of reducing the risk of developing invasive and metastatic disease. Serrano *et al*. (2019) present an overview of therapeutic prevention with a focus on breast and colorectal cancer.

Currently, population‐based screening programmes for early detection are used for cervical, breast, and colorectal cancer. The diagnostic procedures are vital for identifying the relevant tumours and avoiding overtreatment. Dillner (2019) summarizes screening of cervical cancer and suggests how to increase the effectiveness of the processes involved.

Treatment of invasive and metastatic disease at an early stage of the disease increases the cure rate. Early detection is the point of intersection for prevention and therapeutics, and Januszewicz and Fitzgerald (2019) present the development of diagnostic methods for identifying relevant early disease and treatment possibilities using colorectal cancer as a model.

Bridging the gap between research and health care is critical. The CCC integrates cancer care and prevention with research and education, as part of an overall mission to innovate across the whole cancer continuum. Oberst presents an accreditation program for CCCs, integrated centres which have responsibility for multidisciplinary cancer care and quality control by clinical registries. Quality assurance of CCCs is fundamental for institutional collaborations that are required to cover the complete research continuum (Oberst, 2019).

A CCC can serve a population of up to 4–5 million inhabitants, but the rather wide geographical distribution of CCCs implies that not all patients in the surrounding geographic area can be treated at the CCC. Therefore, a CCC needs to organize outreach to local hospitals, and this should be included in the accreditation methodology. In this way, it will be possible to serve all patients, and offer high‐quality, innovative multidisciplinary treatment and care, as described by Brandts (2019).

Survival and cure is one aspect of treatment outcome, but health‐related quality of life (HRQL) is another. HRQL is becoming increasingly important given the growing number of long‐term survivors, both with no evidence of disease or with chronic disease. Due to long‐term physical and psychosocial side effects, cancer survivorship is an increasing human, social, and medical problem. Lagergren *et al*. (2019) describe issues related to cancer survivorship and stress the need of structuring this research area.

Cancer treatment is causing economic problems for health care, and not a single country will be able to cover future costs given current trends. Health economics is currently a missing component that should have a crucial role in assessing the cost‐effectiveness of innovations. It provides health care services with the information required for prioritization to make cancer care cost‐effective, as discussed by Jönsson and Sullivan (2019).

Research‐driven innovation in therapeutics and prevention is dynamic, and education has a primary role to play. Training is needed to increase the quality of translational research, to support the development of CCCs, and to decrease inequalities between EU countries, as argued by Ernberg (2019).

‘Nothing about us, without us’, are critical words from the European Cancer Patient Coalition (ECPC), an umbrella organization of disease‐specific patient organizations introduced here by De Lorenzo and Apostolidis. ECPC is actively involved in a large number of international cancer activities with a focus on policy and ethical questions, and has connections with researchers, politicians and the general public (De Lorenzo and Apostolidis, 2019).

In summary, a well‐prepared cancer community has outlined the issues that need to be addressed in concert with policymakers to implement a mission‐oriented approach to cancer. Prevention can in the long‐term substantially decrease incidence and mortality. Early detection programmes based on innovative diagnostic technologies can stop cancer early by using already proven interventions. Therapeutics will improve survival and quality of life of cancer patients over a shorter time span, thanks to more efficient personalized/precision treatments with anticancer agents, immunotherapy (including cell‐based and vaccination therapies), radiation therapy, surgery, and optimized multimodal treatments. In addition, methodologies for predicting therapy effects will help us abandon ineffective or unnecessary treatments. The positive trends of improved survival (De Angelis *et al*., 2014) and reduced mortality (Carioli *et al*., 2017; Rosso *et al*., 2018; Siegel *et al*., 2019) will gain in strength, and structuring outcomes research will enable rational and cost‐effective implementation in the healthcare system. An ambitious but realizable goal is the long‐term survival of 3 out of 4 cancer patients by 2030 in countries with a well‐developed healthcare system.

## Conflict of interest

Michael Baumann:

In the past 5 years, Dr Baumann attended an advisory board meeting of MERCK KGaA (Darmstadt), for which the University of Dresden received a travel grant. He further received funding for his research projects and for educational grants to the University of Dresden by Teutopharma GmbH (2011?2015), IBA (2016), Bayer AG (2016?2018), Merck KGaA (2016?2030), Medipan GmbH (2014?2018). Dr Baumann, as former chair of OncoRay (Dresden) and present CEO and Scientific Chair of the German Cancer Research Center (DKFZ, Heidelberg), signed/s contracts for his institute(s) and for the staff for research funding and collaborations with a multitude of companies worldwide. For the German Cancer Research Center (DKFZ, Heidelberg) Dr Baumann is on the supervisory boards of HI?STEM gGmbH (Heidelberg).

For the present study, Dr Baumann confirms that none of the above mentioned funding sources were involved in the study design or materials used, nor in the collection, analysis and interpretation of data nor in the writing of the paper.

Alexander Eggermont:

AME declares Honoraria over last 5 years for any speaker, consultancy or advisory role from: Actelion, Agenus, Bayer, BMS, CellDex, Ellipses, Gilead, GSK, HalioDX, Incyte, IO Biotech, ISA pharmaceuticals, MedImmune, Merck GmbH, MSD, Nektar, Novartis, Pfizer, Polynoma, Regeneron, RiverDx, Sanofi, Sellas, SkylineDx.

For the present study, Dr. Eggermont confirms that none of the above mentioned funding sources were involved in the study design or materials used, nor in the collection, analysis and interpretation of data nor in the writing of the paper.
